# Physiological and Transcriptomic Characterization of Sea-Wheatgrass-Derived Waterlogging Tolerance in Wheat

**DOI:** 10.3390/plants11010108

**Published:** 2021-12-30

**Authors:** Wenqiang Li, Ghana S. Challa, Ajay Gupta, Liping Gu, Yajun Wu, Wanlong Li

**Affiliations:** Department of Biology and Microbiology, South Dakota State University, Brookings, SD 57007, USA; wqli@nwafu.edu.cn (W.L.); challa.ghanashyam@gmail.com (G.S.C.); Ajay.gupta@sdstate.edu (A.G.); liping.gu@sdstate.edu (L.G.); yajun.wu@sdstate.edu (Y.W.)

**Keywords:** hypoxia response, nitrate reduction, nitric oxide, sea wheatgrass, waterlogging tolerance, wheat

## Abstract

Waterlogging, causing hypoxia stress and nitrogen depletion in the rhizosphere, has been an increasing threat to wheat production. We developed a wheat–sea wheatgrass (SWG) amphiploid showing superior tolerance to waterlogging and low nitrogen. Validated in deoxygenated agar medium for three weeks, hypoxia stress reduced the dry matter of the wheat parent by 40% but had little effect on the growth of the amphiploid. To understand the underlying mechanisms, we comparatively analyzed the wheat–SWG amphiploid and its wheat parent grown in aerated and hypoxic solutions for physiological traits and root transcriptomes. Compared with its wheat parent, the amphiploid showed less magnitude in forming root porosity and barrier to radial oxygen loss, two important mechanisms for internal O_2_ movement to the apex, and downregulation of genes for ethylene, lignin, and reactive oxygen species. In another aspect, however, hypoxia stress upregulated the nitrate assimilation/reduction pathway in amphiploid and induced accumulation of nitric oxide, a byproduct of nitrate reduction, in its root tips, and the amphiploid maintained much higher metabolic activity in its root system compared with its wheat parent. Taken together, our research suggested that enhanced nitrate assimilation and reduction and accumulation of nitric oxide play important roles in the SWG-derived waterlogging tolerance.

## 1. Introduction

Waterlogging or soil flooding, leading to the depletion of oxygen (hypoxia) [1] and nitrogen nutrients in the rhizosphere [2], has been an increasing threat to crop production as climate change projects more floods [3]. It adversely affects ~10% of arable land worldwide, and 15–20% of the 70 million hectares sown to wheat each year [4]. Wheat yield is reduced linearly with the duration of waterlogging, ~2% per day, at the stem elongation stage [5]. In the lower Mississippi Valley, average yield losses of 44% are found in waterlogging field conditions [6]. Thus, waterlogging-tolerant wheat cultivars are urgently needed to minimize yield loss. Unfortunately, the development of waterlogging-tolerant wheat cultivars has been unsuccessful mainly because the level and heritability of waterlogging tolerance in wheat is low [7] and the trait is usually controlled by a large number of quantitative trait loci with small effect [8,9]. Another factor that hinders genetic improvement of wheat waterlogging tolerance is the significant knowledge gaps in understanding the molecular processes that regulate waterlogging tolerance in wheat. Investigations on waterlogging response and tolerance in wheat have been mainly focused on phenotype description and physiological characterization in response to the hypoxia stress. The formation of aerenchyma [10,11,12,13,14] and the barrier to radial oxygen loss (ROL) [12] are two major mechanisms for adaptation to waterlogging stress.

In the last decade, much progress has been made in understanding the physiological and molecular mechanisms and the genetic control of submergence tolerance in the model plants Arabidopsis and rice [15,16]. The rice submerge resistance genes *SUBMERGENCE1* (*SUB1*) [15,17], *SNORKEL1* (*SK1*), and *SK2* loci [16] encode group-VII ethylene response factors (ERFVIIs), and their expression is induced by submergence in leaves [15,16]. Typical ERFVIIs are characteristic of the N-terminal (Nt) sequence motif MCGGAII/L [18]. In the presence of either ambient nitric oxide (NO) or oxygen (O_2_), targeted proteolysis of ERFVIIs by the evolutionarily conserved N-end rule pathway (NERP) is a key mechanism involved in the hypoxic response in plants [19,20]. NERP links the fate of a protein with the identity of its Nt-residue by destabilizing its Nt motif that starts with the amino acid sequence MC [21]. Under hypoxia, thus, ERFVIIs accumulate to coordinately regulate the hypoxia-responsive genes [19,20]. As a result, modification of NERP components and the N-termini of ERFVII members altered the phenotype of hypoxia response [19,20,22,23]. As an atypical ERFVII member [15], SUB1A-1 resists NERP-mediated degradation, partly explaining the enhanced responsiveness to submergence and drought [19]. Similar to rice, ERFVIIs also interact with the GA [24] and ABA [21] pathways in Arabidopsis. In contrast, little is known about the mechanism of plant response to nitrogen depletion under hypoxia stress.

Waterlogging with the shoot in the air, a situation very different from the submergence conditions of the rice stories, can be lethal for dryland crops such as wheat. A huge quantity of genetic variations are harbored in the genomes of the wild relatives of wheat, some of which showed excellent waterlogging tolerance. One of the examples is the identification of waterlogging tolerance in the wild barley (*Hordeum marinum*) [25]. The characterization of the wheat–*H. marinum* amphiploids revealed that waterlogging tolerance is mainly due to the formation of a strong ROL barrier [25]. We recently discovered superior waterlogging tolerance in progenies derived from crosses between wheat and sea wheatgrass (SWG; *Thinopyrum junceiforme*; 2n = 4x = 28; genomes J_1_J_1_J_2_J_2_), a distant relative of wheat and a relatively untapped resource for wheat improvement [26]. The objectives of this study were to shed light on the mechanisms of the SWG-derived waterlogging tolerance by analyzing the wheat–SWG amphiploid and its wheat parent grown in aerated and hypoxic solutions using physiological and transcriptomic approaches. Different from the previous studies, the SWG-derived waterlogging tolerance showed less magnitude in the formation of aerenchyma and ROL barrier but highly enhanced the nitrogen assimilation and accumulation of nitric oxide in the root tips compared with its wheat parent. Our results suggest a complex mechanism underlying the SWG-derived waterlogging tolerance, in which increased nitrogen assimilation and reduction, NO accumulation, and eventually root metabolic activity possibly play major roles. 

## 2. Materials and Methods

### 2.1. Plant Materials and Growth Conditions

We used an amphiploid (Amph819) derived from crosses between a tetraploid wheat parent (Tt139; (*Triticum turgidum* subsp. *dicoccon*; 2n = 4x = 28; genomes AABB) and SWG accession PI 414667 [26]. The amphiploid Amph819 was germinated 5 d before tetraploid wheat parent Tt139 to ensure that plants were at a similar developmental stage at the onset of hypoxia treatment. Seeds were rinsed with deionized water and placed on moist filter paper in Petri dishes at 22 °C under light conditions. After 3 d, well-germinated seeds were selected and grown in plastic baskets half-submerged in the full-strength nutrient solution [26] in a greenhouse room set at 22 °C at day (16 h) and 16 °C at night (8 h) for 4 d. Twelve-day-old Amph819 and seven-day-old Tt139 seedlings were then transplanted into a 30 L tray (35 cm height × 600 mm length × 40 cm width) containing 25 L full-strength nutrient solution and grown hydroponically with aeration for 3–4 weeks, 16 plants per tray. Eight plants of each genotype similar in size were selected and transplanted into a new tray containing aerated full-strength nutrient solution (aerated conditions) or stagnant deoxygenated nutrient solution (stagnant conditions). The composition for the nutrient solution at full strength was described by Li et al. [26]. For aerated conditions, full-strength nutrient solution in the tray was circulated and aerated steadily by both water pumps and aerators, which maintained constant dissolved oxygen (DO) concentration at 6.0–6.5 ppm (mg/L). For stagnant conditions (hypoxia treatment), 0.1% agar was added into the nutrient solution to produce a stagnant deoxygenated nutrient solution. The dissolved agar impedes O_2_ re-entry and promotes ethylene accumulation [27]. After plants grew in the stagnant deoxygenated nutrient solution for 1 d, the DO level in the solution was checked to be below 1.5 ppm using a DO meter (Gain Express Holdings Ltd., Hongkong). After 3 d of growth in the stagnant deoxygenated nutrient solution, its DO level was checked to be 0.5–1.0 ppm. These nutrient solutions were renewed every 10 days. Two weeks after the hypoxia treatment, the secondary roots were collected for anatomical and histochemical characterization. 

### 2.2. Morpho-Anatomical Characters

To determine the root and shoot length, tiller number, the number of secondary roots, and plant dry mass, plants were sampled at three and six weeks after hypoxia treatment. In principle, an equal number of only healthy plants (8 plants for each sample) were sampled for evaluation and data analysis. For the measurement of root porosity, root segments of about 50 mm in length were prepared at distance from 10 mm to 60 mm from the root tips of the secondary roots. Root porosity was measured by a pycnometer method and calculated using the formula described by Yang, et al. [28]. ROL was detected using the methylene blue agar method as described by Martin et al. [29]. Methylene blue was prepared at 13 mg L^−1^ in a deoxygenated solution containing 0.1% agar, and sodium dithionite (Na_2_S_2_O_4_) was added at 130 mg L^−1^ to reduce the methylene blue so that it was colorless. For the convenience of visualization, 3–4 secondary roots per plant with a length of 80 mm or greater were selected, and all other roots were trimmed off immediately before use. Plants were transferred to the methylene blue solution in a glass tube (diameter 4.5 cm; length: 29.5 cm) and held with the root–shoot junction positioned at 50 mm below the surface of the solution. The tube was incubated at 22 °C under the white light inside the room. After 30 min, the staining patterns of methylene blue around the roots were pictured. For detection of lignin, secondary roots were stained for 30 min with phloroglucinol/HCl (1% (*w*/*v*) phloroglucinol in 20% HCl) as described by Xu et al. [30]. Lignified tissues or sections were stained red/orange under white light as acidic phloroglucinol produces a red product with cinnamyl aldehyde groups present in lignin. For microscopic observation, root cross-sections were prepared and mounted in phloroglucinol/HCl for 5 min and photographed using a BX53 microscope (Olympus Corporation, Tokyo, Japan).

### 2.3. Root Metabolism

The redox indicator 2,3,5-triphenyl tetrazolium chloride (TTC) was used to examine the root metabolic activity as described by Yamauchi et al. [31]. TTC was dissolved in 0.1 M sodium phosphate buffer (pH 7.0) to a final concentration of 0.6% (*w*/*v*). To aid visualization, 6–7 secondary roots per plant with a length of 60 mm or greater were selected, and all other roots and shoots were trimmed off before use. Roots and root–shoot junctions were completely immersed in the TTC solution and incubated at 37 °C for 90 min. After staining, the roots were photographed with a camera. 

Endogenous nitric oxide (NO) levels were detected by DAF-2 DA (4,5-diaminofluorescein diacetate) following the procedure described by Vicente et al. [32]. Root tips were immersed in 1 mL of 10 mM MES-KCl buffer (pH 6.1), and then 1 μL of 10 mM DAF-2 DA solution was added. After incubating 15 min in the dark, the roots were washed 3 times with deionized water and the fluorescence was examined using a fluorescence microscope as described above. NO intensity was determined by selecting equal areas of the same root zone and analyzing with the Image J software (https://imagej.nih.gov/ij/, accessed on 5 November 2021). 

Reactive oxygen species (ROS) production in root was detected via histochemical staining with 3,3′-diaminobenzidine (DAB) and nitrotetrazolium blue chloride (NBT) as described by Daudi and O’Brien [33] and Kumar, et al. [34], respectively. Fluorescence of 2′,7′-dichlorodihydrofluorescein diacetate (H_2_DCFDA), a cell-permeable fluorogenic probe, was also used to measure ROS production as described by Xu et al. [30]. Briefly, root tips were immersed in 1 mL of 10 mM MES-KCl buffer (pH 6.1), and then 1 μL of 10 mM H_2_DCFDA solution was added. After incubating for 8 min in the dark, the roots were washed 4 times with deionized water, and the fluorescence from the H_2_DCFDA was recorded using a BX53 fluorescence microscope (Olympus Corporation, Tokyo, Japan) with a GFP-4050A filter (EX466/40, EM525/50, DM495, BrightLine^®^).

Ethylene was measured following the method described by Hattori et al. [16]. Briefly, the aerial parts were excised from seedlings, and the remaining underground parts (roots) were placed in a container with a saturated NaCl solution. The gas in the container was deaerated with a vacuum pump, and the gas released from the roots was collected in a test tube using a funnel. The collected gas was transferred to a gas chromatography vial, and the vial was fitted with a rubber stopper while held upside down in a saturated NaCl solution. Then, the vial was tightened and righted up, an aliquot of the headspace gas in the vial was withdrawn with a syringe, and the ethylene content was measured by GC-MS (Agilent 890A/5975C) coupled with CP7348 column (Agilent PoraBOND Q 25 m × 250 μm × 3 μm) with Pulsed Split mode at 20:1 ratio at a flow rate 0.8 mL min^−1^ using hydrogen as a carrier gas. The GC program was initiated at 32 °C held for 4 min and ramped at 110 °C/min to reach 232 °C. The scanning mass range of MSD was between 10 and 50 m/z. The ethylene standard (Airgas, Radnor Township, PA, USA) was used as a positive control with C_2_H_4_/air concentration of 10,000, 625, 39.1, 2.44, and 0.15 ppm (*v*/*v*). Ripening banana fruits were chopped and used as a positive control. Four biological replicates were used for each genotype/treatment combination, and a total of 16 root samples were analyzed.

### 2.4. RNA-Seq Analysis

For the RNA-seq experiment, seedlings of the two genotypes were grown hydroponically with aeration for 6 weeks and then transplanted into a new tray containing aerated nutrient solution (aerated treatment) or stagnant deoxygenated nutrient solution (hypoxic treatment) for 5 d. For each treatment, RNA was isolated from about 30 root tips (each ~1cm in length) pooled as a biological replicate using a Tri-reagent, which was further purified using a Direct-Zol RNA Miniprep Plus Clean & Concentrator^TM^ kit (Zymo Research, Irvine, CA, USA). Twelve RNA samples (two genotypes × two treatments × three samples per genotype) were submitted to Novogene Inc. (Sacramento, CA, USA) for constructing 12 paired-end (PE) RNA-seq libraries and producing ~20 million pairs of 150 bp reads per library for Tt139 and ~40 million reads for Amph819 on the HiSeq2000 sequencing platform (Illumina, San Diego, CA, USA). The processing of the raw reads was conducted following the procedure described by Challa and Li [35]. The transcript abundances were calculated by the RNA-seq quantification program Kallisto [36] using the International Wheat Genome Sequencing Consortium (IWGSC) reference genome assembly RefSeqv2.0 and the gene models v2.0 [37,38]. The differential gene expression analysis was carried out using the edgeR [39]. Briefly, the log-transformed transcript abundances were normalized using the trimmed mean of M-values method, and the differentially expressed genes (DEGs) were identified by comparing the transcript abundances of stagnant condition samples vs. the aerated condition samples from Tt139 and Amph819. The DEGs with at least two-fold change and a *p*-value of <10^−3^ were selected for annotations using the BLASTp against the NCBI-nr database, Interpro, and the GO term annotations as described by Challa and Li [35]. The GO terms were enriched against the total GO terms in the genome and filtered by adjusted *p*-value of 0.05. The RNA-seq data were uploaded to BioProject and are available with the accession number PRJNA777361. 

### 2.5. Statistical Analysis

Data were compared using the Duncan’s multiple range test (5% á) and presented as the mean ± standard deviation. 

## 3. Results

### 3.1. Effect of Hypoxic Stress on Morpho-Anatomical Characters

**Figure 1:**
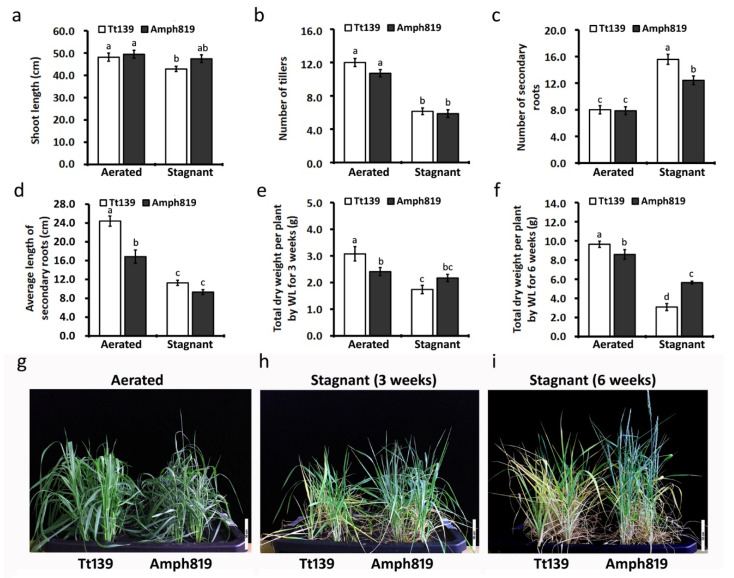
Plant growth and phenotypes of Tt139 and Amph819 seedlings under aerated and stagnant conditions. (**a**–**d**) Shoot length (**a**), the number of tillers (**b**), the number of secondary roots (**c**) per plant, and the average length of secondary roots (**d**) were measured from Tt139 and Amph819 seedlings under aerated and stagnant conditions 3 weeks after treatment. (**e**,**f**) The dry mass of Tt139 and Amph819 seedlings grown in aerated or stagnant conditions for 3 weeks (**e**) and 6 weeks (**f**), respectively. Values are means ± SE (*n* = 8). Letters indicate significant differences between means, determined using Duncan’s multiple range test (5% α). (**g**–**i**) The phenotypes of Tt139 and Amph819 grown in aerated condition (**g**) and stagnant condition for 3 weeks (**h**) and 6 weeks (**i**).

Compared with the controls grown in the aerated solution, three weeks of hypoxic treatment significantly inhibited the shoot growth of the wheat Tt139, whereas it did not affect the amphiploid Amph819 (Figure 1a,h). While the hypoxic stress significantly decreased the number of tillers per plant (Figure 1b), it promoted the production of secondary roots (Figure 1c) in both Tt139 and Amph819. The average length of secondary roots under hypoxic stress was decreased by ~50% in both Tt139 and Amph819 (Figure 1d). As a result, the 3-week hypoxic treatment significantly reduced the total biomass of Tt139 to ~60% but did not cause a significant reduction in Amph819 (Figure 1e,g,h). As the hypoxia treatment entered the 6th week, both Tt139 and Amph819 had a significant reduction in biomass (Figure 1f), but the reduction was more severe in Tt139 than Amph819, which was supported by better survival rate and reproductive growth in Amph819 (Figure 1i). In fact, after 6 weeks of the hypoxic treatment, most leaves of Tt139 had visibly wilted, and the plants were almost dead; by contrast, the Amph819 plants remained alive and began to head and flower (Figure 1i).

We further investigated the effect of waterlogging by subjecting Tt139 and Amph819, as well as rice plants (cultivar Nipponbare), to more severe hypoxia stress. Unlike other cereal crops, most rice cultivars are highly tolerant to low-oxygen conditions or waterlogging. After 8 weeks of hypoxic treatment, the Tt139 plants were completely wilted and dry, the growth of Amph819 plants was significantly repressed, but the rice plants still grew as healthy as the controls under the aerated solution (Figure S1a,b). In the hypoxic treatment, the three genotypes showed a highly variable root system, which was dead and rotten in Tt139, still alive but severely repressed in Amph819 (Figure S1c,d), and healthy in rice (Figure S1c). These results demonstrated that the waterlogging tolerance of Amph819, compared with Tt139, depends mainly on the viability of the root system under hypoxic stress. Thus, we focused on characterization of root traits and transcriptomes under the aerated and hypoxia conditions.

### 3.2. Effect of Hypoxic Stress on Root Porosity

**Figure 2:**
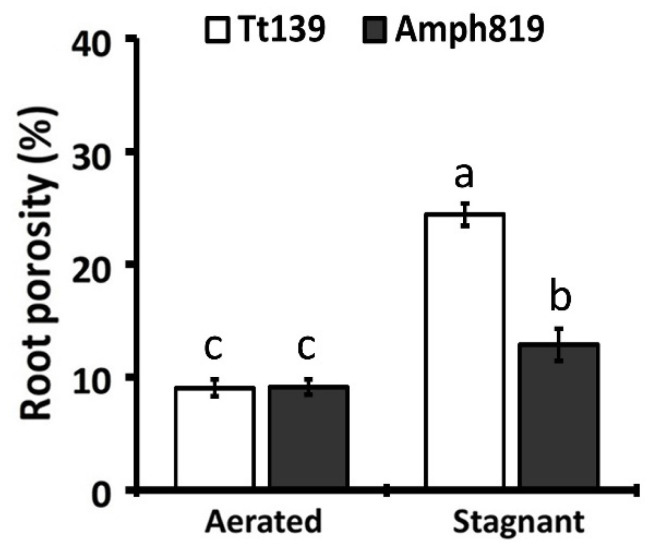
Root porosity (%, total gas spaces in roots) for secondary roots. Tt139 and Amph819 seedlings were grown in aerated or stagnant conditions for 2 weeks. Values are means ± SE (*n* = 5). Letters indicate significant differences between means, determined using Duncan’s multiple range test (5% α).

In many plants, an increase in root porosity is considered an important feature of plant response to hypoxic stress. Thus, we measured root porosity in Tt139 and Amph819 two weeks after the onset of the hypoxic treatment. In aerated conditions, the root porosity in both Tt139 and Amph819 was less than 10% and did not show significant difference between the two genotypes. The hypoxic treatment increased root porosity in Tt139 to 24.4%, which was significantly greater than the 12.9% in the Amph819 (Figure 2). Thus, the net increase in root porosity in the waterlogging-sensitive Tt139 is fivefold as high as that in the waterlogging-tolerant Amph819.

Considering that root porosity is mainly contributed by ROS-triggered aerenchyma formation [30,40], we analyzed the responses of ROS in root tips to the hypoxia stress by DAB and NBT histochemical staining and H_2_DCF-DA fluorescent staining. The ROS level was decreased in both Tt139 and Amph819 after 2 weeks of hypoxia treatment, but no significant difference was observed between the two genotypes (Figure S2).

### 3.3. Effect of Hypoxic Stress on ROL, Lignin Deposition, and Ethylene Level in Roots

**Figure 3:**
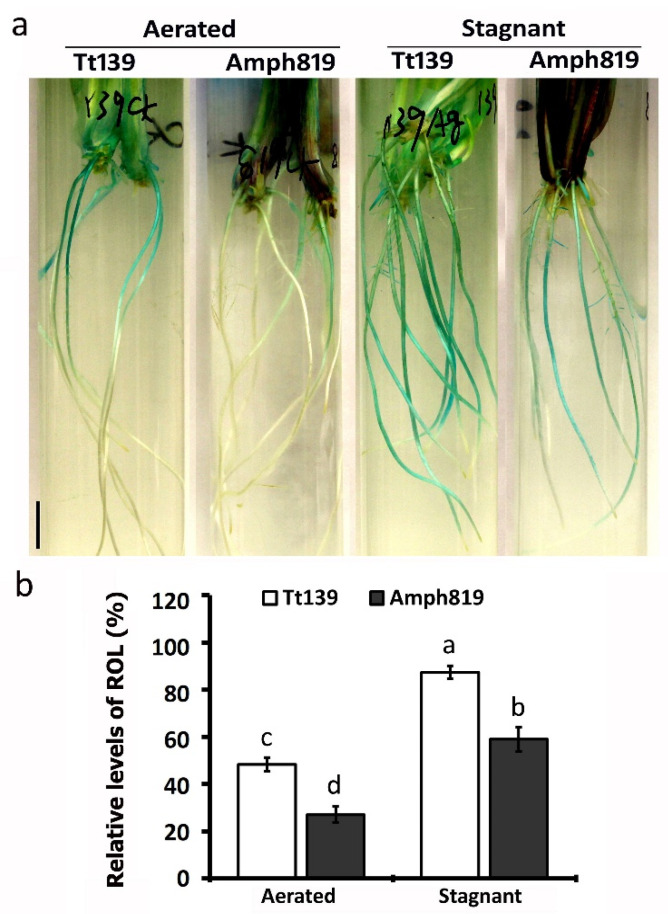
Patterns of ROL along with secondary roots of Tt139 and Amph819 under aerated and stagnant conditions. (**a**) ROL from secondary roots visualized by methylene blue staining in an oxygen-free medium. The seedlings were grown under aerated and stagnant conditions for 2 weeks. The blue color indicates oxygen that has diffused outward from roots. Bars = 1 cm. (**b**) Relative level of ROL. These data are based on the ratio of methylene blue staining (blue staining length/root length). Values are means ± SE (*n* = 16) from more than 6 plants in each treatment. Letters indicate significant differences between means, determined using Duncan’s multiple range test (5% α).

In many plants, the formation of the barrier to ROL is an important mechanism underlying waterlogging tolerance. To determine if the barrier to ROL plays a role in the SWG-derived waterlogging tolerance, we first examined the ROL using methylene blue staining of secondary roots of Tt139 and Amph819. In the aerated condition, Tt139 showed an approximately two-fold higher level of ROL compared with Amph819 (Figure 3a,b). The hypoxic stress increased the ROL level in both Tt139 and Amph819, but the ROL level was still significantly lower in Amph819 (Figure 3a,b). 

**Figure 4:**
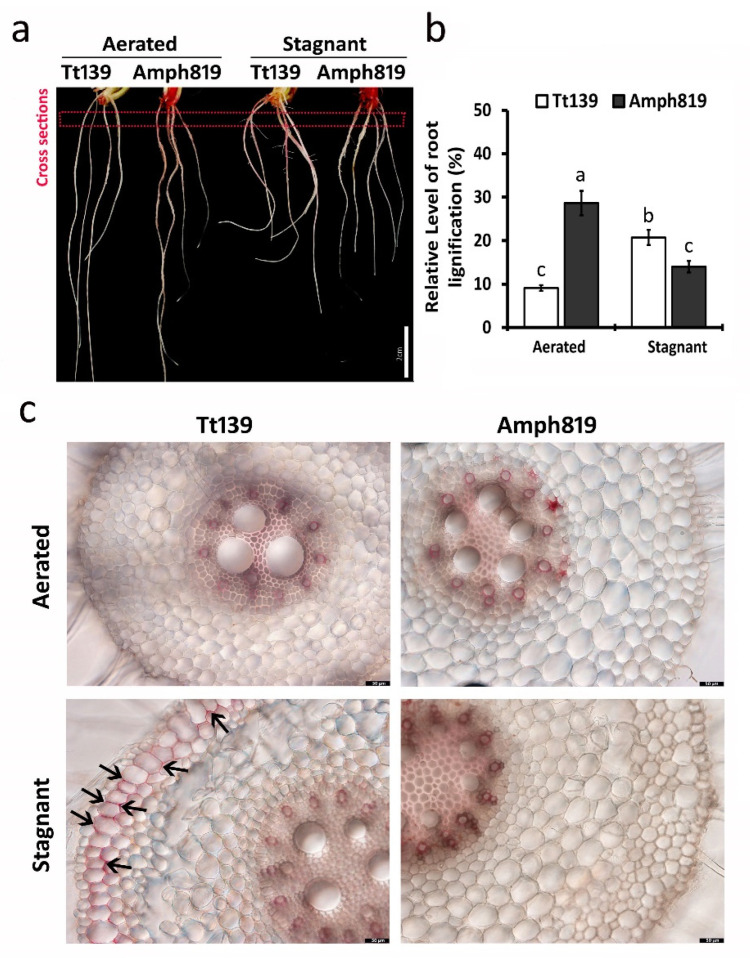
Patterns of secondary roots lignification in seedlings grown under aerated and stagnant conditions for 2 weeks. (**a**) Secondary roots were stained by 1% phloroglucinol-HCl for 30 min. The red staining on roots indicates the deposition of lignin. (**b**) The relative level of root lignification. These data are based on the ratio of lignin staining length (red color)/root length. Values are means ± SE (*n* = 6) from 3 plants in each treatment. Letters indicate significant differences between means, determined using Duncan’s multiple range test (5% α). (**c**) Cross-sections stained by phloroglucinol-HCl for 5 min and viewed under white light. Lignin in cell walls was detected by red staining. Arrows indicate the deposition of lignin in exodermis cells. Scale bars = 2 cm (**a**), and 50 μm (**c**).

Because the barrier to ROL is thought to be lignin deposited at the outer cellular space (apoplast) in the outer part of roots [41], we further examined the lignin deposition in secondary roots by phloroglucinol staining. We found that lignin deposition is mainly located in the region away from the root tip (Figure 4a). In the aerated condition, the Amph819 showed approximately a three-fold higher level of lignin staining than that of Tt139 (Figure 4b). The converse was observed in the two-week stagnant treatment, which increased the lignin deposition by more than two-fold in the Tt139 and reduced it by about 50% in Amph819. We then examined lignin deposition in the root cross-sections in both treatments. In the aerated condition, both Tt139 and Amph819 deposited lignin mostly in the vascular tissues around the stele of roots (Figure 4c and Figure S3). After hypoxia treatment, increased lignin deposition was also found in the outer cellular space of root exodermis in Tt139 (Figure 4c and Figure S3). In the Amph819 roots, however, increased lignin deposition after hypoxia treatment was not detected in the outer part of roots but the vascular bundles (Figure 4c and Figure S3). 

Considering the important role played by ethylene in response to hypoxia stress in previous studies, we examined the level of ethylene in the roots of the aerated and stagnant conditions using GC-MS. We detected ethylene from ripening banana fruit as a positive control but did not detect ethylene in all 16 wheat root samples (four samples for each of the two genotypes and two treatments) (Figure S4), indicating that the ethylene levels in wheat roots are below the limit of detection on our GC-MS system.

### 3.4. Transcriptomic Analysis of Root Tips

The results of root porosity (Figure 2), ROL (Figure 3), lignin (Figure 4), and ethylene cannot explain the out-performance of Amph819 over Tt139 in stagnant treatment (Figure 1). To gain insights into the molecular mechanisms of the SWG-derived waterlogging tolerance, we profiled the transcriptomes of root tip samples of the hypoxia-stressed and aerated control of the Amph819 and the tetraploid wheat parent Tt139. An average of approximately 30 million reads per Tt139 sample and approximately 54 million reads per Amph819 sample were obtained after removing the contamination and low-quality reads (Table S1). Because both genotypes contain the identical A and B genomes, we mapped the clean reads to the annotated A and B genome of the common wheat (*T. aestivum*) cv. Chinese Spring reference genome and identified 7191 differentially expression genes (DEGs; 2983 upregulated and 4208 downregulated) in Amph819 and 5643 DEGs (2959 upregulated and 2684 downregulated) in Tt139 (Figure S5). Comparison of the DEG profiles identified 3747 DEGs (1796 upregulated and 1951 downregulated) shared by the waterlogging-tolerant Amph819 and waterlogging-sensitive Tt139. These 3747 DEGs are probably involved in the basal response to hypoxic stress (Table S2). Removal of the shared DEGs identified 3444 (1187 upregulated and 2257 downregulated) DEGs unique to Amph819 and 1896 (1163 upregulated and 733 downregulated) DEGs unique to Tt139 (Table S3). Of these genotype specific DEGs, seven genes were upregulated in Amph819 but downregulated in Tt139, and 27 genes showed reverse patterns (Table S3). 

DEG enrichment analysis identified diverse biological processes, molecular functions, and subcellular compartments in response to the hypoxia treatment (Tables S4–S7), indicating the complication at the cellular and molecular levels. Many DEGs encode TFs, epigenetic machinery, protein degradation machinery, protein kinases, peroxidases, cytochrome P450, glycosyltransferases, lipases, and transporters (Table S3), indicating that hypoxia reshapes transcriptome, proteome, metabolome, and the cellular redox status in the root tips. One of the most significant changes upon hypoxic treatment is the downregulation of 132 histone-coding genes in Amph819 but only five were downregulated in Tt139 (Table S8), suggesting that cell division activity in root tips of Amph819 is dramatically decreased probably for energy conservation. Consistent with this, genes coding for transcription factors Scarecrow and WUSCHEL-related homeobox proteins, which are important for root meristem maintenance, were downregulated in Amph819 but upregulated in Tt139 (Table S8). 

Protein kinases, calcium, phytohormones, and TFs are important components of the signal transduction networks. Nine mitogen-activated protein kinase (MAPK) genes were upregulated three- to ten-fold in Amph819 (Table S8), indicating that the MAPK pathway plays a role in hypoxia response. A total of 100 genes functioning in phytohormonal biosynthesis and/or signaling pathways were differentially expressed between Amph819 and Tt139 in response to the stagnant treatment, of which 96 are involved in auxin, brassinosteroid, ethylene, gibberellin, and jasmonic acid pathways. Of these 96 genes, 71 biosynthetic and positive signaling genes were downregulated and one negative regulator upregulated in Amph819. Twenty-two biosynthetic and positive signaling genes were upregulated and three catabolic genes downregulated in Tt139 (Table S8). Out of the 100 genes, on the contrary, three genes positively regulating abscisic acid (ABA) signaling pathways were downregulated, and one gene encoding protein phosphatase 2C (PP2C), a negative regulator of ABA signaling, was upregulated in Tt139 (Table S8). 

**Figure 5:**
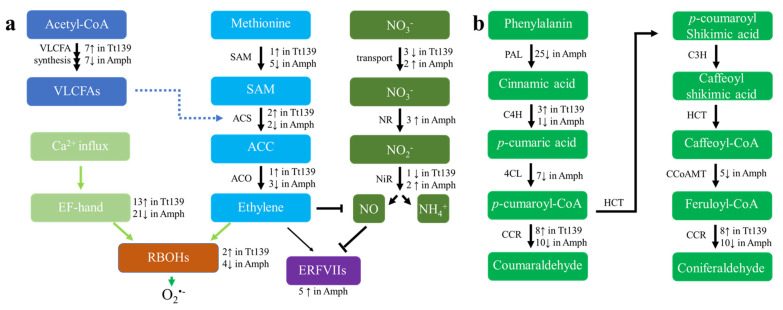
Genetic pathways and differentially expressed genes in root tips in response to hypoxia stress. (**a**) Part of the VLCFA, ethylene, ROS, and NO biosynthesis pathways and (**b**) part of the lignin biosynthetic pathway are presented with enzymes or proteins indicated at the left and numbers of the transcripts upregulated (↑) or downregulated (↓) by stagnant treatment in Tt139 and Amph819 (Amph) indicated at the right. In the genetic pathways, arrows between components indicate stimulation of activities at protein (bold arrows) or transcription level (fine arrow), and the T-bars indicate inhibition. The fold change of the transcripts can be found in Table S8.

Among these phytohormones, ethylene is believed to play a central role in hypoxic response and submergence tolerance in the model plants [42,43]. It is proposed that sub-oxygen condition promotes the transcription of ethylene biosynthetic genes, enhanced ethylene synthesis induces transcription of genes encoding respiratory burst oxidase homologs (RBOHs), and activation of RBOHs by EF-hand protein kinases-mediated phosphorylation leads to the production of ROS, which triggers the formation of lysigenous aerenchyma [40]. Transcription of genes involved in this pathway is downregulated by the hypoxia treatment in Amph819, including 10 ethylene biosynthetic genes, 21 EF-hand domain-containing protein-coding genes, and four *RBOHs* (Figure 5a; Table S8). In contrast, transcription of five ethylene biosynthetic genes, 13 EF-hand genes, and two *RBOHs* were upregulated in Tt139 under hypoxia treatment (Figure 5a; Table S8). In addition, an ethylene receptor *ETR2* gene that negatively modulates ethylene-activated signal, was upregulated in Amph819 due to the hypoxia treatment (Table S8). This result indicates that the ethylene signal is depressed in root tips of the waterlogging tolerant Amph819.

Different from the expression state of the ethylene biosynthetic genes, five ERFVII genes were upregulated in Amph819 by two- to four-fold (Figure 5a; Table S8). Outstanding of the TF DEGs, 17 WRKY genes are downregulated by 2- to 93-fold in Amph819, and two WRKY genes were upregulated in Tt139 (Table S8), suggesting that these WRKY TFs may have a negative effect on hypoxia tolerance. In addition, four genes coding for MADS-box protein *Vrn1* and its homologs, which showed a pleiotropic effect on root development and growth [44], were upregulated in Amph819 (Table S8).

Lignin is a major component of the ROL barrier [41]. The hypoxic treatment downregulated the expression of 56 lignin biosynthetic genes in Amph819, including 7 for arogenate dehydrogenase, 25 for phenylalanine ammonia-lyase (PAL), 7 for 4-coumarate CoA ligase (C4L), 1 for cinnamate 4-hydroxylase (C4H), 5 for caffeoyl-CoA O-methyltransferase (CCoAMT), and 10 for cinnamoyl CoA reductase (CCR) but increased expression of 3 C4H and 8 CCR genes in Tt139 (Figure 5b; Table S8). These genes encode enzymes required for seven reaction steps of the lignin monomer synthesis. Most dramatic changes were found in the CCoAMT, CCR, and PLA genes (Table S8). In Arabidopsis, AtMYB63 positively regulates these lignin biosynthetic genes [45]. Three wheat genes homologous to AtMYB63 were downregulated six- to ten-fold by hypoxia stress in Amph819 (Table S8). These results are consistent with the lignin staining (Figure 4).

### 3.5. Nitrogen Assimilation and Nitric Oxide Accumulation in Root Tips under Hypoxia Stress

Hypoxia stress not only causes energy crisis but nitrogen deficiency as well [1]. Previously, we found that Amph819 exhibited increased tolerance to low nitrogen [26], implying a possible association of nitrogen metabolism changes to waterlogging tolerance. Using our transcriptome data mining, we found two high-affinity nitrate transporter genes, three nitrate reductase (NR) genes, and two nitrite reductase (NiR) genes that were upregulated in Amph819 upon hypoxic stress (Figure 5a; Table S8). In contrast, one high-affinity nitrate transporter gene, two dual-affinity nitrate transporter genes, and a nitrite reductase gene were downregulated in Tt139 (Figure 5a; Table S8). The same nitrite reductase gene is upregulated in Amph819 (Table S8).

**Figure 6:**
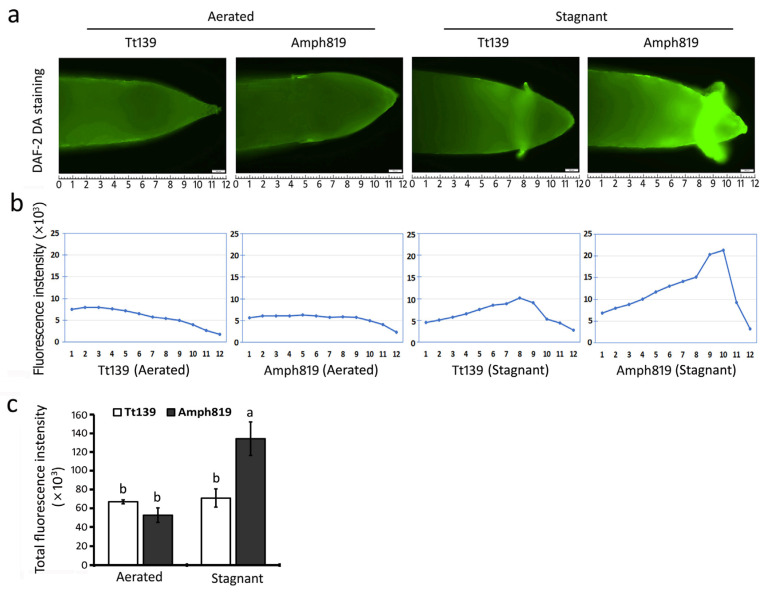
Fluorescent detection of nitric oxide (NO) in root tips of seedlings grown under aerated and stagnant conditions for 2 weeks. (**a**) Detection of NO accumulation by fluorescent staining with DAF 2-DA in root tips. Genotypes and growth conditions are indicated on the top. (**b**) Fluorescence intensity at the corresponding position of the root tip. The numbers underneath indicate the positions from the root tips corresponding to that in (**a**). (**c**) Total fluorescence intensity of the root tips. Values are means ± SE (*n* = 4). Letters indicate significant differences between means, determined using Duncan’s multiple range test (5% α).

In addition to reducing nitrate to ammonia, NR/NiR activity involves the production of nitric oxide (NO), an important metabolite and signaling molecule in many plants and tissues [46]. We, therefore, examined NO accumulation in root tips by fluorescent staining with DAF 2-DA, a fluorescent NO probe. Under the aerated conditions, NO level was very low in the roots of both Tt139 and Amph819 (Figure 6a,b); under the stagnant conditions, hypoxia stress-induced high level of NO accumulation in the root cap of Amph819 but not in that of Tt139 (Figure 6a,b). Statistical analysis showed about 2-fold increases in Amph819 but no increase in Tt139 (Figure 6c), suggesting a possible association of the increased NO accumulation with the SWG-derived waterlogging tolerance.

### 3.6. Root Metabolic Activity under Hypoxia Stress

**Figure 7:**
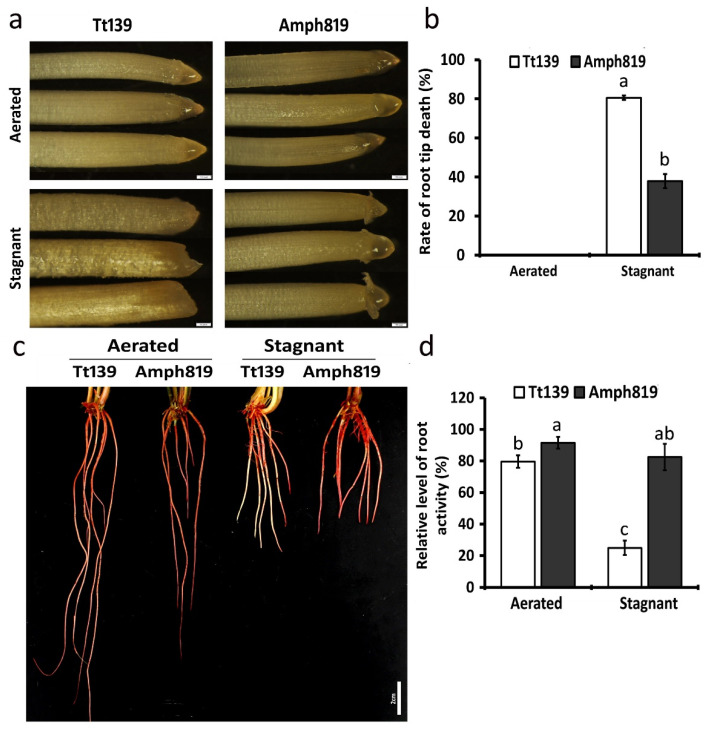
Effect of hypoxic stress on root activity. (**a**) Phenotypes of Tt139 and Amph819 root tips under aerated and stagnant conditions for 3 weeks. Scale bars = 200 μm. (**b**) Rate of root tip death. (**c**) TTC staining of secondary roots grown in aerated and stagnant conditions. The red color indicates the TTC is reduced by dehydrogenases in living cells. Scale bar = 2 cm. (**d**) Relative Level of root activity. These data are based on the ratio of root staining length (red color)/root length by TTC staining. Values are means ± SE (*n* = 12) from 3 plants in each treatment. Letters indicate significant differences between means, determined using Duncan’s multiple range test (5% α).

As a potent oxidant and electron recipient, NO is expected to influence root metabolic activity under hypoxia stress. With this, we analyzed the secondary root activity under stagnant conditions. The hypoxia stress caused the death of the secondary root tips, where the root apical meristem is located. The initial root tip/cap death and decay were observed in Tt139 after two weeks of stagnant treatment, but it occurred much later in Amph819. The death rate of root tips at the end of the third week of the stagnant treatment was more than 80% in Tt139 whereas smaller than 40% in the Amph819 (Figure 7a,b). We also examined the root metabolic activity by staining with TTC, which is normally colorless but turns red when reduced by dehydrogenases in living cells. TTC staining showed that the Tt139 lost root activity after three weeks of hypoxia treatment, which was evident from the low staining along the root length, particularly near the root tip compared with the well-stained Amph819 in most parts of the root length (Figure 7c). Hypoxia stress reduced the TTC staining level to ~30% in Tt139 roots but rarely changed the staining level in Amph819 (Figure 7d). 

## 4. Discussion

Waterlogging results in anoxia in soil and hypoxia in roots, which forces a shift of energy metabolism in roots from aerobic respiration to fermentation [47]. To cope with the energy crisis created by hypoxia, plants adopt an array of strategies to survive the hypoxic stress, including the formation of aerenchyma to increase O_2_ diffusion from shoots and the formation of a ROL barrier to preserve O_2_ in root. Aerenchyma is formed through ROS-induced programmed cell death [10,30] and contributes the root porosity, and the ROL barrier is formed via the deposition of lignin and suberin in the outer layers of the roots [41]. While the induction of aerenchyma formation is a common phenomenon in many plants for adapting to hypoxia, the ROL barrier is drawing recent attention as an important mechanism underlying the hypoxia tolerance, which was transferred from wild relatives into wheat [25] and maize [41]. We detected the increased root porosity upon the stagnant treatment in both genotypes but at a much lower level in the waterlogging tolerant Amph819 compared with the waterlogging sensitive Tt139 (Figure 2), suggesting that the induced root porosity is an adaptative response to hypoxia stress but did not significantly contribute to the SWG-derived waterlogging tolerance. Our data showed that a lower level of root porosity paralleled the lower level of ROL in Amph819 compared with Tt139. Root porosity is contributed by ROS-triggered aerenchyma formation [30,40]. The lower level of root porosity is consistent with the downregulation of the *RBOHs* (Figure 5a; Table S8) although no significant difference of ROS was detected between Tt139 and Amph819 (Figure S2). Regarding the ROL barrier, the stagnant treatment did increase lignin deposition in exodermis in Tt139 but generally reduced the lignin deposition in Amph819, consistent with the dynamic expression of lignin biosynthetic genes (Figure 5b; Table S8). The increased lignin deposition in Tt139 is seemingly not enough to protect the increased O_2_ input due to the increased root porosity (Figure 2 and Figure 3). In the model plants Arabidopsis and rice, submergence elevates the ethylene level which induces transcription of the downstream genes, and eventually leads to enhanced survival under hypoxic stress. We did not detect ethylene in the stagnant-treated roots in both Amph819 and Tt139 using the method adopted from rice research. Other more sensitive methods, such as newly developed fluorescence probes [48], need to be used for the detection of ethylene in hypoxia-stressed roots. Transcriptome analysis, however, showed that the ethylene signal is depressed by the hypoxia stress in Amph819 but induced in Tt139 (Figure 5a; Table S8), implying that ethylene did not significantly contribute to the SWG-derived waterlogging tolerance. All these results indicate that waterlogging-sensitive Tt139 had a stronger response to the hypoxia stress than the waterlogging-tolerant Amph819. The only gene expression pattern consistent with phenotype is the downregulation of the 132 histone genes and upregulation of five ERFVII genes (Table S8). Because ethylene biosynthetic genes were downregulated in Amph819 during hypoxia stress (Figure 5a; Table S8), the increased transcription of these *ERFVIIs* is probably independent of the ethylene signal and different from Arabidopsis, where most of the *ERFVIIs* are induced by ethylene [49]. The increased ERFVII proteins, however, could be repressed by the NERP triggered by the accumulated NO in the root tips (Figure 6).

An important but largely neglected aspect is the nitrogen deficiency caused by waterlogging [1,2]. In addition to the superior waterlogging tolerance, Amph819 also showed excellent tolerance to low nitrogen [26]. In this study, we detected upregulation of genes functioning in the nitrate assimilation/reduction pathway in Amph819 compared with Tt139. In tomatoes, hypoxia activates NR by promoting disassociation from inhibitor protein 14-3-3 and dephosphorylation [50]. Thus, the upregulated transcription of the nitrate assimilation/reduction genes including NR may explain less severity of nitrogen deficiency symptom (leaf-yellowing) in the former (Figure 1h,i) although more work is needed to measure NR activity in the roots and the nitrogen content in the shoots. 

Translocation of nitrogen from medium to shoots requires a functional root system, which is supported by the apical meristem activity. In this respect, Amph819 maintained a much higher level of metabolic activity in roots compared with its wheat parent Tt139 (Figure 7). Where is the cellular energy (ATP) from to sustain the root metabolic activity in Amph819 under the hypoxia stress? Because upregulation and/or downregulation of the fermentation pathway were found in both genotypes, additional energy sources may underlie the root metabolic activity in Amph819. One of the sources would be NO-enhanced respiration. As an O_2_-derived free radical molecule, NO plays an important role in many physiological processes, including the response of plants to hypoxia [42]. In maize, the chemical block of the hypoxia-induced NO burst significantly impaired the survival of root tips [51]. Our current knowledge suggests that NO functions in hypoxia response in two aspects. First, NO may regulate the respiration enzymes, such as cytochrome c oxidase and aconitase, via nitrosylation, thus tightening the regulation of respiration and oxygen consumption [52,53,54]. Second, anoxic reduction of nitrate to NO leads to the generation of some ATP and recycles NAD(P)H to NAD(P)^+^ via the phytoglobin/NO cycle, and the regenerated NAD(P)^+^, in turn, fuels up glycolytic fermentation for more ATP production [55,56]. The NO burst occurs a few hours upon hypoxia and then declines to the prior level even though hypoxic stress continues [42]. The increased NO accumulation, however, was maintained in the root tips of Amph819 grown in the agar-containing stagnant solution for two weeks (Figure 6). Thus, the NO detected in the root tips of Amph819 is more likely to function as a metabolite in one or both respects. In the model plant Arabidopsis, ethylene mediates the depletion of NO [49]. The downregulation of ethylene biosynthetic genes in Amph819 by the stagnant treatment probably facilitated the NO accumulation in its root tips (Figure 6). Taken together, upregulation of the nitrate assimilation and reduction not only provide nitrogen nutrition to shoot but also generate NO as a byproduct, which, in turn, could have contributed to the energy production to maintain the viability of the root tips and root activity of the wheat-SWG amphiploid. Validation of this notion will open the door for improving waterlogging tolerance by engineering the nitrate assimilation and reduction pathway and by use of the germplasm of high nitrogen use efficiency.

We are dissecting the SWG genome in the wheat background by crossing and backcrossing the amphiploid to wheat cultivars and observed waterlogging tolerance in the progenies carrying one or two SWG chromosomes. We are also assembling the SWG genome sequences. The improved SWG genome assembly will be used to analyze the RNA-seq data from the amphiploid and identify the DEGs from the SWG genome. These resources can be used to validate the results from the present study. 

## Data Availability

The data presented in this study are available in Supplementary Materials and BioProject accession number PRJNA777361.

## Supplementary Materials

The following are available https://www.mdpi.com/article/10.3390/plants11010108/s1. Figure S1. Comparison of waterlogging tolerance among Tt139, Amph819, and rice plants. Figure S2. Detection of ROS accumulation in roots by histochemical and fluorescent staining. Figure S3. Patterns of secondary roots lignification in seedlings grown under aerated and stagnant conditions for 2 weeks. Figure S4. GC-MS analysis of the gases released from the roots did not detect ethylene. Figure S5. Volcano plots of differentially expressed genes upon stagnant treatment. Table S1. The number of clean reads. Table S2. Differentially expressed genes shared by Amph819 and Tt139 upon hypoxia stress. Table S3. Unique differentially expressed genes detected. Table S4. GO classification of the upregulated DEGs in Amph819 upon stagnant treatment. Table S5. GO classification of the downregulated DEGs in Amph819 upon stagnant treatment. Table S6. GO classification of the upregulated DEGs in Tt319 upon stagnant treatment. Table S7. GO classification of the downregulated DEGs in Tt319 upon stagnant treatment. Table S8. Differentially expression genes putatively contributed to SWG-derived hypoxia tolerance in wheat.
