# Supplementary material for: Physiological and Transcriptomic Characterization of Sea-Wheatgrass-Derived Waterlogging Tolerance in Wheat
Source: Plants (Basel). 2021 Dec 30;11(1):108. doi: 10.3390/plants11010108 (PMC8747256; doi:10.3390/plants11010108)
Supplement: Supplementary file 1 [file plants-11-00108-s001.zip › plants-1475305-supplementary figures for XML.pdf]

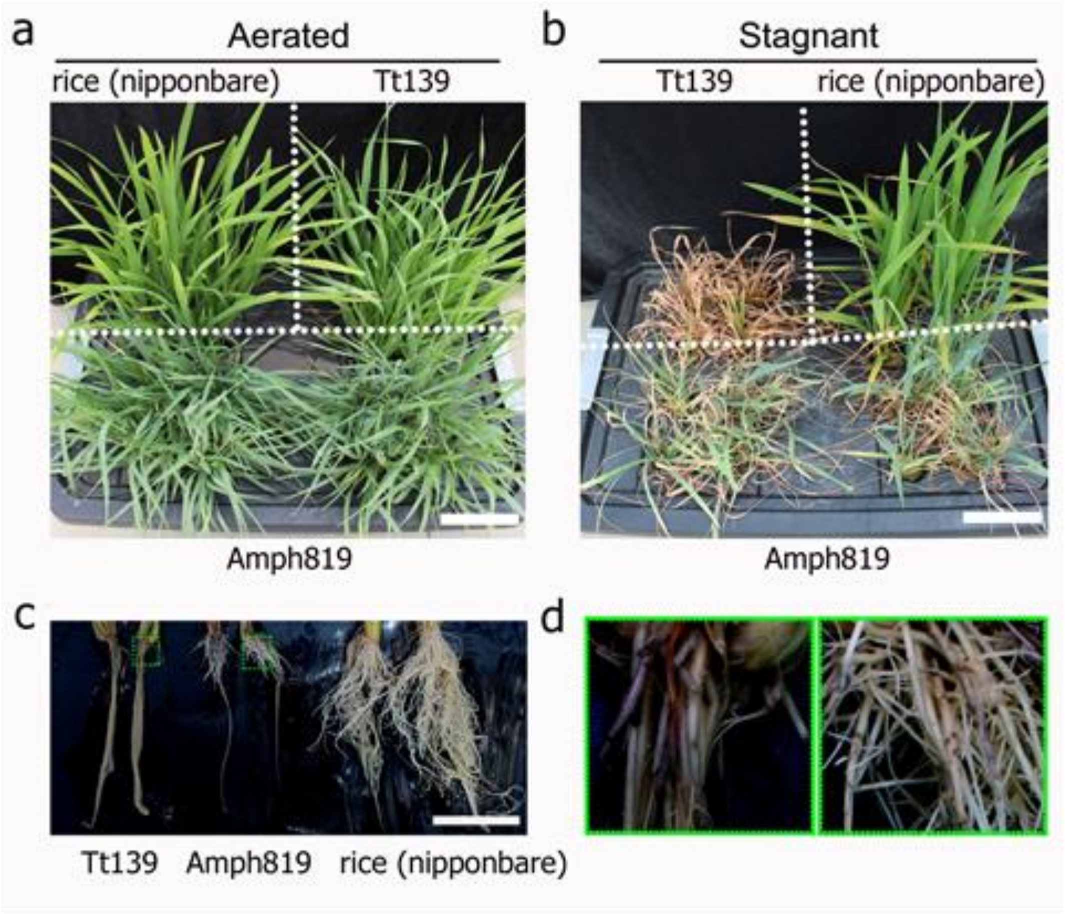

Figure S1. Comparision of waterlogging tolerance among Tt139, Amph819 and rice plants. (**a**,**b**) The seedlings of Tt139, Amph819 and rice Nipponbare grown in aerated condition (**a**) and stagnant condition (**b**) for 8 weeks. (**c**) The root system of Tt139, Amph819 and Nipponbare rice plants grown in the stagnant condition. (**d**) A partial enlarged view of Tt139 (left) and Amph819 (right) root system grown in the stagnant condition.

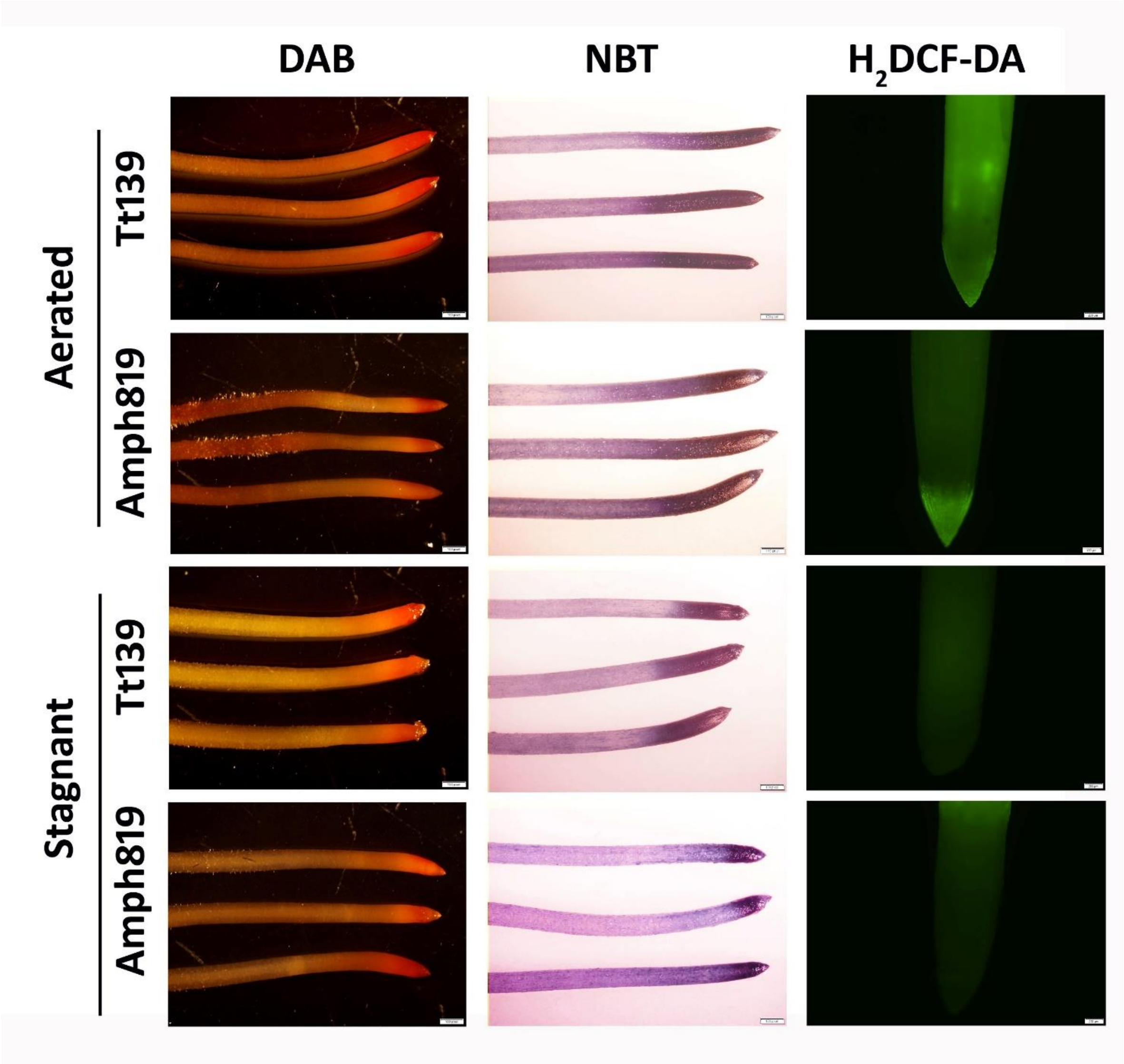

Figure S2. Detection of ROS accumulation in roots by histochemical and fluorescent staining. The Tt139 and Amph819 seedlings were grown in aerated and stagnant conditions for 2 weeks. Secondary roots were stained by DAB, NBT, and H_2DCF-DA. Scale bars = 500μm for DAB and NBT staining, 200μm for H_2DCF-DA staining.

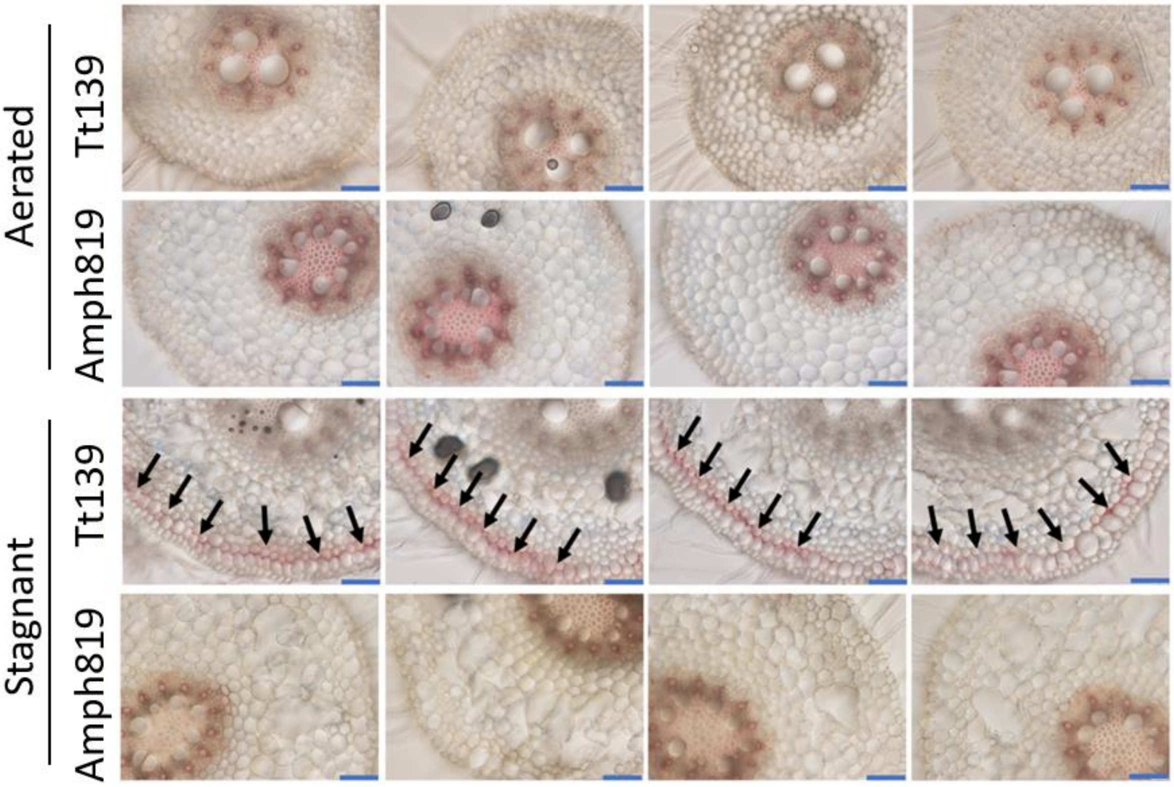

Figure S3. Patterns of secondary roots lignification in seedlings grown under aerated and stagnant conditions for 2 weeks. Lignin staining were conducted in cross sections of root base of Tt139 and Amph819 grown under aerated and stagnant conditions for 2 weeks. Bars =100 μm.

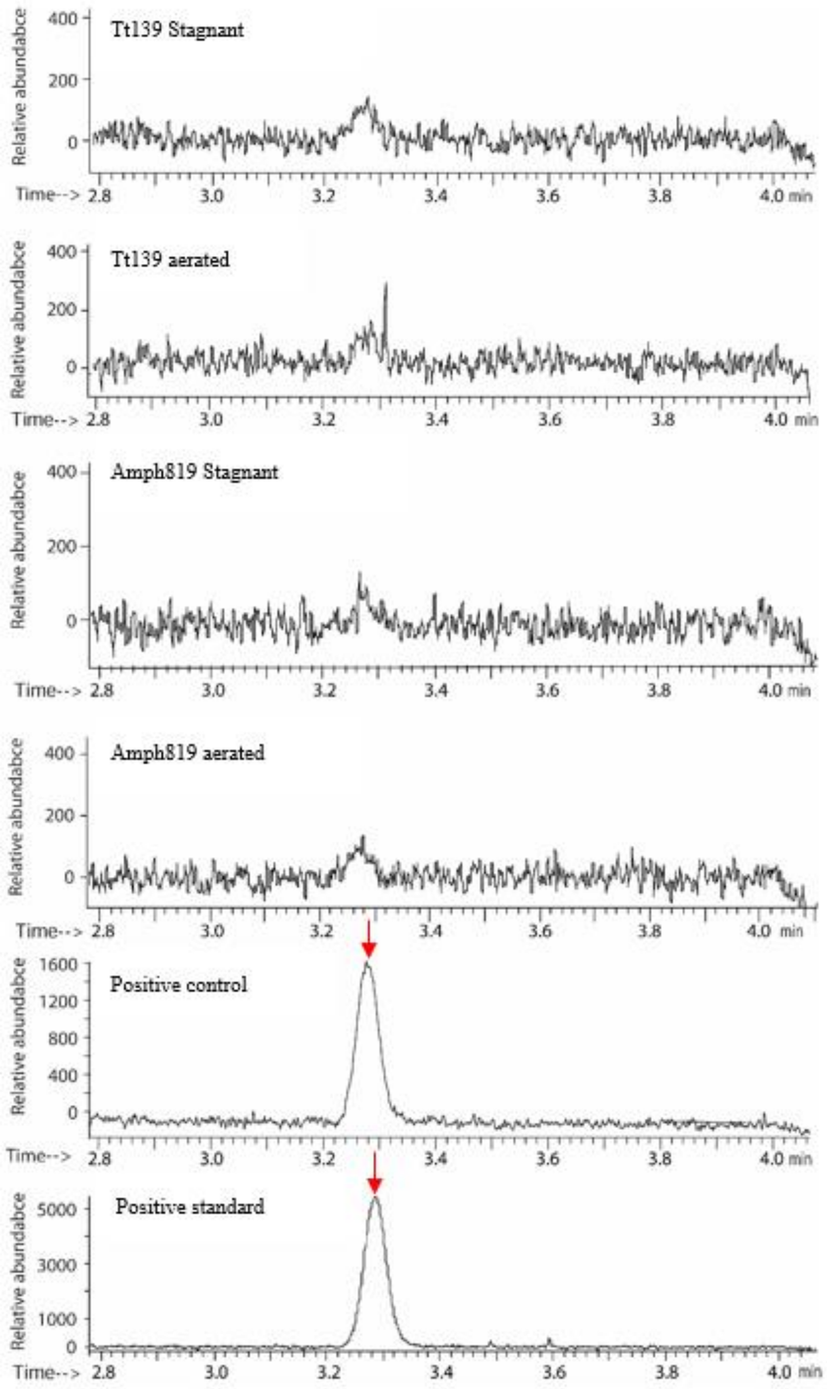

Figure S4. GC-MS analysis of the gases released from the roots did not detect ethylene. Sample names are indicated in the left upper corner of profiles. The numbers in the x-axes indicate the minutes of gas chromatographing. The numbers on the y-axes indicate relative abundance of substances. The arrows indicate the position of ethylene in the positive control and positive standard. The concentration of ethylene in the positive control was 39.1 ppm concentration.

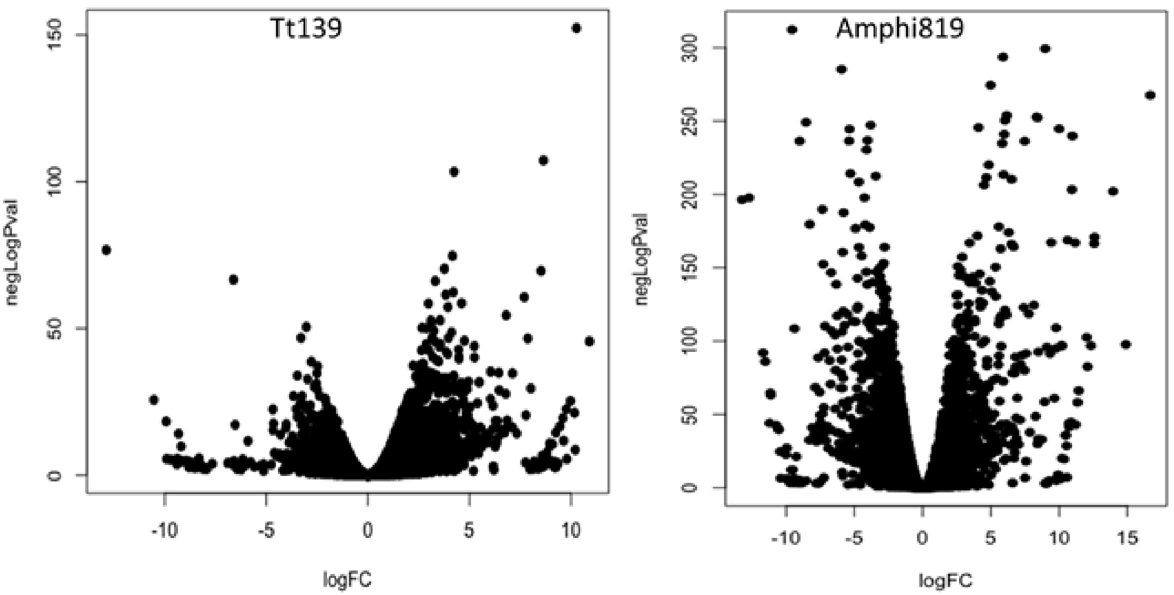

Figure S5. Volcano plots of differentially expressed genes upon stagnant treatment. The numbers on the x-axes are the log_2-converted fold change in the stagnant-treated root tips compared to the aerate ones. The numbers on the y-axes are the negative log_{10}-converted p values. The genotypes are indicated on the top.
